# Left Atrial Remodeling in Response to Aortic Valve Replacement: Pathophysiology and Myocardial Strain Analysis

**DOI:** 10.3390/life12122074

**Published:** 2022-12-10

**Authors:** Matteo Lisi, Maria Concetta Pastore, Alessio Fiorio, Matteo Cameli, Giulia Elena Mandoli, Francesca Maria Righini, Luna Cavigli, Flavio D’Ascenzi, Marta Focardi, Andrea Rubboli, Gianluca Campo, Sergio Mondillo, Michael Y. Henein

**Affiliations:** 1 Department of Medical Biotechnologies, Division of Cardiology, University of Siena, 53100 Siena, Italy; 2 Department of Cardiovascular Disease—AUSL Romagna, Division of Cardiology, Ospedale S. Maria Delle Croci, Viale Randi 5, 48121 Ravenna, Italy; 3 Institute of Public Health and Clinical Medicine and Heart Centre, Umeå University, 90187 Umeå, Sweden; 4 Cardiology Unit and LTTA Centre, University of Ferrara, 44121 Ferrara, Italy

**Keywords:** aortic stenosis, replacement, remodelling, left atrial strain, speckle tracking

## Abstract

Severe aortic stenosis (AS) is the most common valve disease in the elderly and is associated with poor prognosis if treated only medically. AS causes chronic pressure overload, concentric left ventricular (LV) hypertrophy, myocardial stiffness, and diastolic dysfunction. This adverse remodeling also affects the left atrium (LA), which dilates and develops myocardial fibrosis, with a reduction in intrinsic function and a consequent high risk of the development of atrial fibrillation. Speckle-tracking echocardiography is able to detect myocardial dysfunction before other conventional parameters, such as LV ejection fraction, and also predict clinical outcomes. This review aims at describing LV and LA remodeling in AS and before and after aortic valve replacement and the usefulness of myocardial strain analysis in this clinical setting.

## 1. Introduction

Aortic stenosis (AS) is the most common heart valve disease both in Europe and North America [1]. Its incidence is increasing due to the aging of the population, reaching a prevalence of 4–5% in individuals over 65 years [2,3]. The most common cause of AS is degeneration and calcification of the leaflets, irrespective of the valve anatomy, tricuspid or bicuspid. This etiology accounts almost for 80% of cases in Western Europe, followed by rheumatic disease, which is characterized by commissural fusion with leaflet retraction and is more common in less industrialized countries [4].

The normal aortic valve area is >2 cm^2^. In AS, the valve opening gradually decreases and the degree of stenosis becomes severe with a valvular area lower than 1 cm^2^ [5]. The resulting obstruction causes a pressure overload on the left ventricle (LV) with the development of concentric myocardial hypertrophy as an adaptive mechanism to reduce wall stress. Early in the disease process, such changes lead to diastolic dysfunction due to the reduction of LV compliance, while systolic performance is only mildly compromised. Later on, myocardial contractile function and deformation become impaired and, consequently, cardiac output falls. These changes can easily be followed by the accurate recording of trans-valvular Doppler velocities and pressure drop, which both drop as the systolic function becomes significantly compromised. The left atrium (LA) also shares some of these cardiac morphological changes, with its size increasing due to chronic pressure overload, which eventually leads to an increase in pulmonary venous and arterial pressure [6], with resulting heart failure (HF) symptoms development and, often, atrial fibrillation (AF) [7].

The prognosis of patients with untreated symptomatic severe AS is poor, affecting approximately 80% of patients within 4 years of the onset of clinical manifestations [8]. AS has therefore become the most frequent indication for surgical and percutaneous intervention among structural heart diseases. According to the latest ESC/EACTS guidelines for the management of valvular disease, intervention for severe AS is indicated, irrespective of symptoms, in patients with one of these conditions, either isolated or associated: systolic dysfunction in absence of other causes; severe AS with mean gradient ≥ 60 mmHg; rapid progression and markedly elevated BNP levels, particularly in those with estimated poor prognosis [9]. The type of intervention should be decided based on the heart team evaluation. Surgical aortic valve replacement (SAVR) remains the first choice in patients <75 years of age with low surgical risk, while transcatheter aortic valve replacement (TAVR) is preferred in those >75 years with high surgical risk [9]. SAVR provides greater guarantees regarding prosthesis durability and has lower rates of permanent pacemaker implantation and vascular complications. On the other hand, TAVR is associated with a lower probability of bleeding, acute kidney injury, and shorter hospital stay. Another important difference between the two approaches is the rate of new-onset AF, which is lower in TAVR, because of the lower impact of the procedure on LA, with reported respective probability of 5–20% and 30–60% for developing AF [10,11,12].

LA and LV remodeling can be noninvasively assessed with echocardiography. In the last years, speckle-tracking echocardiography (STE) has emerged as an easy, quick, and advanced tool to provide early detection of myocardial functional and structural properties. Much research showed cardiac chamber remodeling anticipating the changes in basic echocardiographic parameters, with important diagnostic and prognostic implications.

The aim of this review is to provide information on the pathophysiology of left atrial remodeling after SAVR and TAVR in patients with AS and to provide an overview of the increasing evidence on the use of speckle-tracking echocardiography to detect these changes. A literature review was conducted to identify English language studies in Pubmed from 2009 to 2022, using combinations of the following search terms: ‘left atrial,’ ‘remodelling’ or ‘remodeling’, ‘aortic valve replacement’, ‘aortic valve repair’, ‘aortic valve implantation’, ‘speckle-tracking echocardiography’, ‘myocardial strain’. Examination of reference lists of these studies yielded additional studies. Studies were reviewed by two independent authors and then used for the first paper draft, which was implemented and reviewed by all the authors.

## 2. Left Atrial Remodeling and Myocardial Fibrosis in Aortic Stenosis

LV and LA function are closely related; in fact, the latter modulates LV filling and plays an important role in the diastolic phase of the cardiac cycle, while LV function influences LA contraction and relaxation. This interdependence means that changes in the intracavitary pressures of one chamber also affect the other [13].

**Figure 1:**
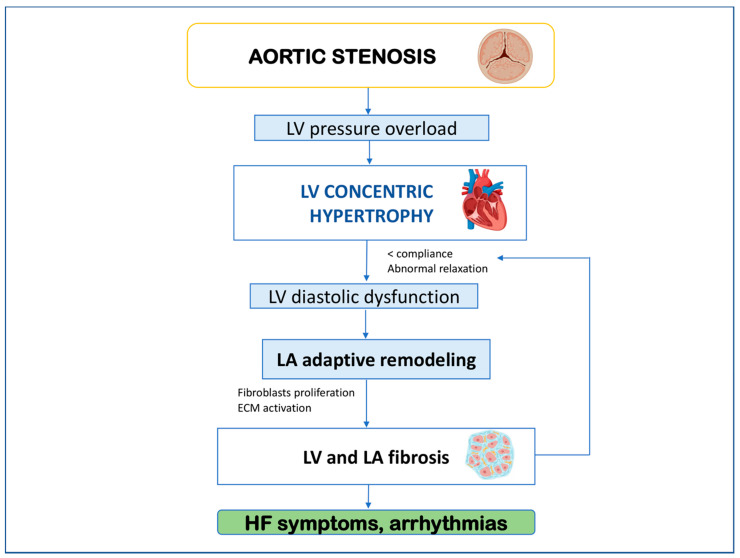
Mechanism underlying clinical deterioration in aortic stenosis. ECM, extracellular matrix; HF, heart failure; LA, left atrium; LV, left ventricle.

In AS, systolic pressure overload leads to compensatory concentric LV hypertrophy to preserve normal wall stress and EF [14]. Wall thickening causes diastolic dysfunction due to decreased compliance and impaired relaxation, which requires raised filling pressures in order to maintain normal end-diastolic volume. This is reflected in the LA, which undergoes adverse remodeling [15]. These hemodynamic adaptations determine excessive activation of cardiac fibroblasts with resulting collagen formation. Consequently, the progressive extracellular matrix (ECM) expands, and LV and LA myocardial fibrosis develop [16]. This chronic process is first adaptive but progressively results in significant disturbances of myocardial fiber architecture. The enhanced ECM incorporates cardiac myocytes and microvascular cells leading to myofibrils dysfunction, increased wall stiffness, and impaired relaxation [17]. Such LA structural and function changes eventually lead to the deterioration of diastolic function, and HF symptoms and predispose patients to arrhythmias such as AF [Figure 1].

Cardiac magnetic resonance (CMR) with late gadolinium enhancement (LGE) is currently the gold standard imaging technique for the detection and quantification of both focal and diffuse myocardial fibrosis [18]. Gadolinium deposits in extracellular space allow the identification of fibrosis as areas of hyper-intensity on T1-weighted images, which appear a few minutes after contrast infusion. It is a reliable technique that has been validated against myocardial biopsy findings [19]. However, CMR has its known limitations, particularly limited availability, accessibility, and high costs.

More recently, STE has been used to evaluate myocardial function, showing high accuracy in detecting myocardial fibrosis and early LA function disturbances, particularly in patients with preserved LV EF and normal LA volume quantified by two-dimensional measurements [20]. This technique offers a measure of myocardial deformation through the analysis of conventional echocardiographic images acquired on a stable ECG tracing; it is increasingly available on echocardiographic machines as well and may be applied to all cardiac chambers. The use of STE in assessing LA reservoir function, measuring peak atrial longitudinal strain (PALS), proved superior over conventional echocardiographic parameters for the diagnostic and prognostic evaluation of HF with both reduced and preserved EF [21,22] as well as predicting new-onset AF [23,24]. Indeed, several studies have shown the ability of STE in identifying myocardial fibrosis that correlated with histopathological analyses in different settings, such as patients undergoing surgical valve repair or heart transplantation, in whom PALS proved a good predictor of LA fibrosis extension and was associated with clinical outcomes [25,26].

## 3. Left Ventricular and Left Atrial Remodeling after Aortic Valve Replacement

**Figure 2:**
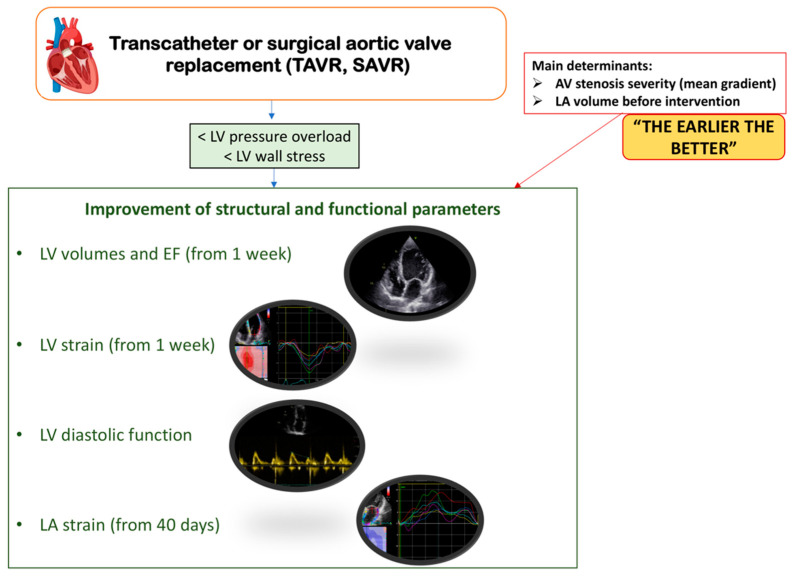
Structural and functional myocardial changes after surgical or transcatheter aortic valve replacement. AV, aortic valve; EF, ejection fraction; LA, left atrium; LV, left ventricle; SAVR, surgical aortic valve replacement; TAVR, transcatheter aortic valve replacement.

Both surgical and transcatheter aortic valve replacement reduce trans-valvular pressure drop (gradient) and increase the aortic valve orifice area. This leads to a reduction in pressure overload and wall stress and, consequently, improvement of structural, systolic, and diastolic LV parameters. Garg V. et al., in their systematic review of changes in myocardial deformation after aortic valve replacement [27], have shown that LV ejection fraction (EF) and global longitudinal strain (GLS) improve one week after surgical replacement of the aortic valve. These rapid changes are mainly due to immediate LV pressure offloading after the removal of the outflow tract resistance [28]. Early changes were seen within a few days of SAVR [29,30,31] and TAVR [32,33]; however, TAVR patients seem to have a greater early improvement in GLS, possibly due to the well-known myocardial stunning and abnormal septal motion related to surgery [Figure 2].

Studies have shown that myocardial recovery demonstrated by strain parameters continues to develop for up to one year after TAVR [34,35] and 17 months for SAVR [36] with evidence of better LV EF recovery after TAVR compared with SAVR [37,38]. These late changes reflect a process of reverse remodeling after the restoration of normal heart mechanics with partial regression of maladaptive changes after the removal of pressure overload. However, there is a lack of histopathological data in the literature to confirm that restoration of functional parameters after aortic valve replacement is also accompanied by real regression of myocardial fibrosis. 

Myocardial reverse remodeling after aortic valve replacement also involves the LA. SAVR and TAVR both result in an improvement of LA atrial function with a reduction in LA volume and an increase in PALS 40 days after intervention, which become stable at the 3-month follow-up [27,39]. The main determinant of this structural and functional recovery is the severity of AS evaluated by trans-valvular pressure drop, which indirectly reflects chronic chamber-pressure overload and the extent of myocardial fibrosis. Moreover, LA dimensions before aortic valve replacement have also been found to correlate with LA structural and functional recovery, suggesting that earlier intervention promotes better myocardial remodeling and, though partial, more likely restoration of atrial function [40]. 

## 4. Clinical Implications 

LA speckle-tracking analysis proved to be associated with clinical outcomes. Galli et al. studied 128 patients with severe AS, a quarter of whom underwent TAVR, and found that an overall PALS lower than 21% was a significant predictor of major adverse cardiovascular events (MACE), unlike LA volume [41]. Other reports showed a modest but significant association with clinical outcomes for PALS as well as preoperative LA volume and for transvalvular pressure gradients [42,43]. 

LA function evaluated by STE proved also to be the strongest predictor of post-operative atrial arrhythmias [44], which are the most frequent complication after cardiac surgery but is not an uncommon finding after the transcatheter approach. A systematic review and meta-analysis by Kawczynski et al. evaluated the correlation between pre-operative transthoracic echocardiography and post-operative atrial fibrillation (POAF) and showed the unique ability of PALS in predicting POAF [45]. Thus, patients’ risk stratification based on the pro-arrhythmic substrate by echocardiographic evaluation (LA strain assessment) should be considered an important tool in planning targeted prevention measures such as pharmacological prophylaxis and careful fluid management both pre- and early post-intervention in patients considered at high risk.

Furthermore, studies have shown that LA strain is one of the major determinants of right heart pressures in patients with severe AS and preserved LV EF [46,47]. This could be a further reason to routinely evaluate LA function in order not to delay the intervention and to prevent the onset of irreversible pulmonary hypertension.

## 5. Speckle-Tracking Echocardiography (STE)—Technical Aspects

The STE technique has been developed on two-dimensional strain echocardiographic imaging and is based on the analysis of standard two-dimensional grey-scale echocardiographic images for myocardial function analysis. The speckle patterns (acoustic backscatter generated by the reflected ultrasound beam) are followed frame by frame by the software, using a statistical approach based on the detection of the best matching area. The displacement of these speckles is expected to follow myocardial movement, tracing a movement curve (negative in case of contraction, positive in case of relaxation), which is assumed to represent myocardial deformation (and is considered as a percentage value [48]). Different curves are traced to represent the movement of different chamber segments, and then the software generates an average curve of the chamber deformation derived from the average of the segmental curves. Sometimes, achieving such precision might not be easy because of limited image acquisition. However, with the development of dedicated software for the LA and the right ventricle as well, the accuracy of this technique has significantly augmented also for lower-quality acoustic windows.

## 6. LA Longitudinal Myocardial Intrinsic Function by STE

**Figure 3:**
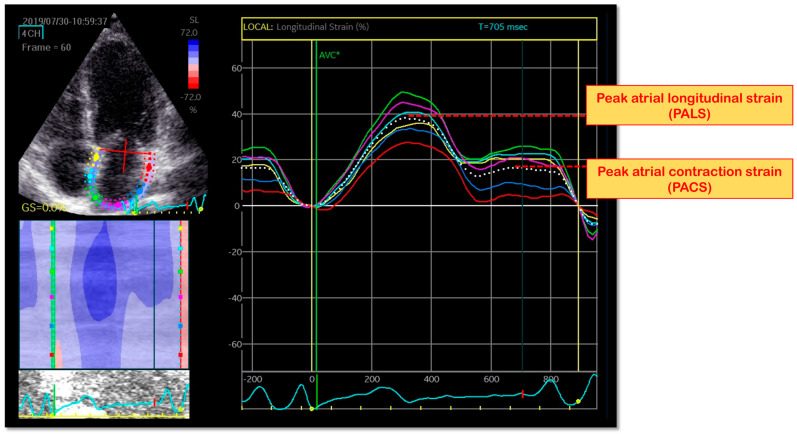
Left atrial strain parameters by speckle-tracking echocardiography.

LA intrinsic myocardial function is obtained using STE from the apical four- and two-chamber views, applying the method described above for LV longitudinal myocardial intrinsic function. Care should always be taken in obtaining true apical images using standard anatomical landmarks to avoid foreshortening the LA, therefore allowing a clear delineation of the LA endocardial border. As previously described [17,49]. LA endocardial border is manually traced in both the four- and two-chamber views, thus delineating a region of interest (ROI), consisting of six segments. After segmental tracking, quality analysis, and manual adjustment of the ROI, the longitudinal strain curves are generated by the software for each atrial segment. Peak atrial longitudinal strain (PALS), measured at the end of the reservoir phase, is calculated by averaging values of all LA segments (global PALS) and by separately averaging values from the four- and two-chamber views. Likewise, peak atrial contraction strain (PACS), obtained during LA contraction, is measured as the average of all 12 segments (global PACS) and by separately averaging values from the two apical views (four- and two-chamber PACS) [50] [Figure 3].

## 7. Left Atrial Strain in Aortic Stenosis

PALS was shown to be correlated with prognosis in patients with several degrees of AS [41,51], and with the development of post-operative AF caused by fibrosis, increased myocardial stiffness, and altered relaxation [44], also regardless of pre-existing LA dilatation [52]. In addition, LA strain was the major determinant of pulmonary hypertension in patients with severe AS and preserved LV EF [47] and a marker of LA remodeling after transcatheter aortic valve replacement [38], suggesting that a serial evaluation of LA function in these patients could help to provide early surgical treatment also in AS before the development of pulmonary hypertension and irreversible LA damage. However, further research is warranted on this topic.

## 8. Conclusions

AS is one of the most frequent valvular heart diseases requiring surgical treatment. Pressure overload due to aortic valve stenosis results in adverse LV and LA remodeling with the development of myocardial fibrosis and diastolic dysfunction with an increased risk of HF and AF. This adaptation is only partially reversible, and recovery is not complete even after aortic valve replacement. The reduction of longitudinal strain evaluated by STE proved to be closely related to myocardial fibrosis and, indeed, is a good predictor of cardiac outcome and the development of atrial arrhythmias. 

The latest ESC guidelines on the management of valvular heart diseases consider invasive treatment of AS based on patient symptoms or LV impairment evaluated by EF but do not consider strain evaluation when deciding on the optimal timing of valve replacement. This strategy often results in late management with irreversible cardiac structural changes. As illustrated in this review, alterations in LV GLS and PALS are able to detect myocardial dysfunction before the reduction of EF and LA enlargement take place. In the near future, their routine use in clinical practice could help identify patients requiring earlier invasive treatment in order to improve post-operative outcomes and recovery. 

## Data Availability

Not applicable.
